# Floral Scent Composition and Fine-Scale Timing in Two Moth-Pollinated Hawaiian *Schiedea* (Caryophyllaceae)

**DOI:** 10.3389/fpls.2020.01116

**Published:** 2020-07-21

**Authors:** John M. Powers, Roger Seco, Celia L. Faiola, Ann K. Sakai, Stephen G. Weller, Diane R. Campbell, Alex Guenther

**Affiliations:** ^1^Department of Ecology and Evolutionary Biology, University of California, Irvine, Irvine, CA, United States; ^2^Terrestrial Ecology Section, Department of Biology, University of Copenhagen, Copenhagen, Denmark; ^3^Center for Permafrost (CENPERM), Department of Geosciences and Natural Resource Management, University of Copenhagen, Copenhagen, Denmark; ^4^Department of Earth System Science, University of California, Irvine, Irvine, CA, United States

**Keywords:** *Schiedea kaalae*, *Schiedea hookeri*, *Pseudoschrankia*, floral volatiles, island flora, moth pollination, gas chromatography - mass spectrometry (GC-MS), proton transfer reaction - mass spectrometry (PTR-MS)

## Abstract

Floral scent often intensifies during periods of pollinator activity, but the degree of this synchrony may vary among scent compounds depending on their function. Related plant species with the same pollinator may exhibit similar timing and composition of floral scent. We compared timing and composition of floral volatiles for two endemic Hawaiian plant species, *Schiedea kaalae* and *S. hookeri* (Caryophyllaceae). For *S. kaalae*, we also compared the daily timing of emission of floral volatiles to evening visits of their shared pollinator, an endemic Hawaiian moth (*Pseudoschrankia brevipalpis*; Erebidae). The identity and amount of floral volatiles were measured in the greenhouse during day and evening periods with dynamic headspace sampling and GC-MS (gas chromatography – mass spectrometry). The timing of emissions (daily rise, peak, and fall) was measured by sampling continuously for multiple days in a growth chamber with PTR-MS (proton transfer reaction mass spectrometry). Nearly all volatiles detected underwent strong daily cycles in emission. Timings of floral volatile emissions were similar for *S. kaalae* and *S. hookeri*, as expected for two species sharing the same pollinator. For *S. kaalae*, many volatiles known to attract moths, including several linalool oxides and 2-phenylacetaldehyde, peaked within 2 h of the peak visitation time of the moth which pollinates both species. Floral volatiles of both species that peaked in the evening were also emitted several hours before and after the brief window of pollinator activity. Few volatiles followed a daytime emission pattern, consistent with increased apparency to visitors only at night. The scent blends of the two species differed in their major components and were most distinct from each other in the evening. The qualitative difference in evening scent composition between the two *Schiedea* species may reflect their distinct evolutionary history and may indicate that the moth species uses several different floral cues to locate rewards.

## Introduction

In flowering plants, attraction of pollinators is often required for reproduction, but the multimodal signals that attract pollinators are costly and require both carbon and energy (Dicke and Sabelis, 1989; Grison-Pigé et al., 2001). Floral signals that attract pollinators may also attract visitors that reduce fitness such as herbivores (e.g. Theis and Adler, 2012; Schiestl, 2015; Nunes et al., 2016), nectar robbers (e.g. Kessler et al., 2008; Kessler and Halitschke, 2009), or generalist pollinators with high heterospecific pollen loads (Morales and Traveset, 2008). Selection on floral signals via pollinators is therefore expected to favor allocation of resources to traits that optimize pollen received or dispersed and minimize costs of apparency to other visitors. When pollinators are active only during a specific time period, temporal regulation of a floral signal is one way to increase efficiency in signaling (Hoballah et al., 2005). For example, the fitness of *Nicotiana attenuata* plants is affected if the timing of flower orientation or olfactory pollination cues is altered physically or genetically (Baldwin et al., 1997; Yon et al., 2017). Overlap between the window of pollinator activity and the timing of floral signals is common, whether the signals are related to physical access (Overland, 1960; Goldblatt et al., 2004), flower orientation (Yon et al., 2017), or scent production (Heath et al., 1992; Huber et al., 2004; Effmert et al., 2005; Kumano and Yamaoka, 2006; Okamoto et al., 2008; Prieto-Benítez et al., 2016; Chapurlat et al., 2018).

These and other previous studies have been useful in identifying the volatiles emitted during a known period of animal activity, for example during the foraging periods of diurnal versus nocturnal pollinators (Bischoff et al., 2014). Knowledge of how closely the time courses of volatile emissions match the activity of a pollinator is still limited, especially since pollinator activity can also change on very short time scales (Herrera, 1990; Knop et al., 2018). Here we generate continuous measurements of volatile emissions to observe the start and end of emissions, so that we can determine if volatiles are emitted outside of the period of pollinator activity and thus at times when costs might exceed benefits for a channel of information for the pollinator. Continuous measurements can also distinguish a volatile that is rising in emission, which might indicate a period of pollinator activity is starting, from a volatile that is declining at a given point in time.

Plant species with the same pollinator might be expected to display similar floral signals, but most tests of floral scent convergence within genera have been restricted to flowers that mimic a female insect (Cortis et al., 2009; Gögler et al., 2009) or oviposition site (Jürgens et al., 2013) or provide a fragrance reward (Nunes et al., 2017). These pollination systems require the presence of key compounds in precise ratios to produce a successful mimic or species-specific pheromone. Food-seeking pollinators may not require such highly specific floral chemical displays. Plant species that reward pollinators with food and have distinct scents might nevertheless attract shared pollinators if pollinators learn to associate the scent of each species with a reward. If heterospecific pollen transfer between related species reduces fitness (by clogging stigmas or producing infertile hybrids), plants would benefit from species-specific signals if distinct scents reduce heterospecific pollen transfer through floral constancy of pollinator individuals (Waelti et al., 2008).

We investigated the composition and timing of floral scent in *Schiedea kaalae* and *S. hookeri* (Caryophyllaceae), two hermaphroditic species with specialized floral nectaries and similar floral morphology (Wagner et al., 2005b) which are pollinated by the endemic Hawaiian moth *Pseudoschrankia brevipalpis* (Erebidae; Weisenberger et al., 2014; Medeiros, 2015; Weller et al., 2017). In this plant genus, wind pollination evolved from biotic pollination (Sakai et al., 2006; Willyard et al., 2011). Reversals from wind to biotic pollination are also possible but cannot be currently verified given the poor resolution of the clade containing nearly all wind-pollinated species as well as several hermaphroditic species, including *S. hookeri* (Willyard et al., 2011). The clades containing *S. kaalae* and *S. hookeri* diverged c. 1.3 Mya (Willyard et al., 2011). Because these species share the same moth pollinator, which visits for a brief period of time in the early evening, we predicted that the two *Schiedea* species would share similar timing of maximum emissions of compounds known to attract moths, but differ in evening floral scent composition due to their separate evolutionary histories.

We first describe the patterns of volatile emissions in these two moth-pollinated species by asking how *S. kaalae* and *S. hookeri* differ in the composition (identity and amount) of evening floral volatile emissions. Next, we characterize how individual volatiles change throughout the day and night in each species. Finally, we quantify the degree of overlap of volatiles (in aggregate and individually) with pollinator activity for one of the species, *S. kaalae*.

## Materials and Methods

### Study System

*Schiedea kaalae* Wawra (sect. *Mononeura*) and *S. hookeri* A. Gray (sect. *Schiedea*) are hermaphroditic, self-compatible, protandrous, perennial herbs native to Oʻahu, Hawaiʻi, USA, where populations of the two species occur in sympatry in parts of the Waiʻanae Mountains [*S. kaalae* (410–730 m above sea level, asl) and *S. hookeri* (260–870 m asl), Wagner et al., 2005b] and can flower at the same time. *Schiedea kaalae* also occurs in the Koʻolau Mountains (Wagner et al., 2005b). Both species are listed as endangered by the US Fish and Wildlife Service and critically endangered by the IUCN (Ellshoff et al., 1991; Bruegmann and Caraway, 2003; Wagner et al., 2005a; Bruegmann et al., 2016), and a total of only about 28 *S. kaalae* individuals in five populations remained in the wild before restoration efforts (Weisenberger et al., 2014), precluding studies of the remnant populations *in situ*. *Schiedea hookeri* is more common in nature than *S. kaalae*, and large populations also exist following restoration efforts (D. Sailer, personal communication). The species produce inflorescences with 20–300 (*S. kaalae*) or 20–150 (*S. hookeri*) flowers per inflorescence and both species possess similar floral morphology with reflexed sepals 3–4 mm long, no petals, 10 stamens, 3 styles, and 5 nectaries (Wagner et al., 2005b).

### Prior Studies of Pollination Biology

The shared moth pollinator of *Schiedea kaalae* and *S. hookeri, Pseudoschrankia brevipalpis* (Weller et al., 2017), perches on flowers (or more rarely, on other parts of the inflorescence) and feeds on nectar extruded from the tips of specialized tubular nectary extensions adjacent to the stamens (Harris et al., 2012; Weisenberger et al., 2014). At ʻĒkahanui Gulch (Waiʻanae Mountains) *P. brevipalpis* was the only visitor to flowers of *S. kaalae*, based on observations over three years (Weller et al., 2017). Fewer pollinator observations were made for *S. hookeri* because of the inaccessibility of the sites, although direct and indirect observations both indicated that *P. brevipalpis* was the primary pollinator at ʻĒkahanui Gulch (Weller et al., 2017). Very low numbers of other endemic moth species were observed visiting *S. hookeri* at a second site and a few carried *Schiedea* pollen, but pollen deposition was threefold lower than at ʻĒkahanui Gulch, and no correlation between moth scales and pollen deposition was observed, indicating the absence of effective pollination (Weller et al., 2017). No daytime floral visitors to either species have been observed (Weisenberger et al., 2014).

The elliptic flight patterns of the moths before they land on flowers suggest they rely little on visual targeting even before dark and are characteristic of moths seeking floral volatiles through anemotaxis (upwind flight; Cardé and Willis, 2008; Weller et al., 2017 and videos therein).

### New Analyses of Field Data for Time of Moth Visits

For comparison with timing of volatile emissions, we determined the timing of flower visits by the moth *P. brevipalpis*. Our earlier studies (Weller et al., 2017) reported the duration of visits to flowers in male and female stages of anthesis but not arrival times. Here we analyzed arrival time of visits (landings on a flower) of *P. brevipalpis* to *S. kaalae* at ʻĒkahanui Gulch (*n* = 48 visits on three consecutive dates in March 2014 and one in July 2014; landings occurred from 17:49–19:28 HAST, 0.2–1.6 h after sunset). Observations of the field population always began at least a half hour in advance of any moth visit and continued until after moth activity ceased, so the entire spectrum of potential arrival times was included. We did not include *S. hookeri* in the analysis of timing of visits because we had too few direct observations of pollinator visits.

Because the timing of moth behavior and floral volatile emission patterns may be driven by light levels (Altenburger and Matile, 1990; Hansted et al., 1994; Hendel-Rahmanim et al., 2007) or circadian rhythms entrained by light cycles (Kolosova et al., 2001; Fenske et al., 2015; Yon et al., 2016; Fenske et al., 2018), we calculated the difference between the times of each moth visit to a flower and local sunset. The angle of elevation to the nearby ridge towards the median solar azimuth at sunset across observation dates was used to determine local sunset, using the *crepuscule* function of the R package *maptools* (Bivand et al., 2019). This technique corrects for the shadows cast by the mountainous terrain. We combined these relative times across dates to create a temporal distribution of moth visits to *S. kaalae*.

### Plants Sampled

Volatile emissions were measured on plants of *Schiedea kaalae* and *S. hookeri* grown in the University of California, Irvine greenhouse. Plants were potted in UC mix (a soil mix developed by the University of California; 1:1:1 sand, peat, and redwood fiber) with added perlite and watered as needed with dilute liquid fertilizer (Grow More; 20-20-20 NPK plus micronutrients). Plants were grown from seeds or cuttings of six populations from the Waiʻanae Mountains (10 *S. kaalae* and 10 *S. hookeri* plants, **Supplementary Table S1**; all collections were made before species were listed as federally endangered in 1991 and 1996, for *S. kaalae* and *S. hookeri*, respectively). Plants also were grown from intraspecific (mostly interpopulation) crosses between cultivated plants from these populations (22 *S. kaalae* plants, 22 *S. hookeri* plants). Interpopulation crosses within species were used because most natural populations now consist of a single individual and are highly inbred (Weisenberger et al., 2014). For GC-MS measures, we sampled 32 plants of each species in the evening (see below). Four *Schiedea kaalae* and eight *S. hookeri* plants from this group were also sampled during the day. For continuous PTR-MS measurements of plants in a growth chamber over multiple days, we sampled five *S. kaalae* plants, two from Puʻumaialau (Takeuchi 3587) and three from Pahole Gulch (Weller and Sakai 904), and three *S. hookeri* plants, one from Kaluaʻa Gulch (Weller and Sakai 879, 400 m south of Puʻuhapapa) and two from Waiʻanae Kai (**Supplementary Table S1**). All plants chosen had ≥ 10 open flowers. The numbers of open male- and female-phase flowers, closed (post-anthesis) flowers, and floral buds were recorded immediately after sampling for both methods. Inflorescence age, as estimated by the ratio of closed to open flowers, did not vary between species in the sampled plants (ANOVA, P = 0.90, *n* = 64).

### Scent Collections and Analysis by GC-MS

#### Scent Collections

Procedures for dynamic headspace sampling for GC-MS were modified from Campbell et al. (2019). Scent traps, consisting of a glass capillary tube filled with 5 mg of Tenax TA and held with plugs of silanized quartz wool, were cleaned before initial use by heating in helium carrier gas for 5 min at 250 °C. Scent samples were collected from November 2016 - April 2017 in the greenhouse during evening and daytime sampling periods. The natural day length varied from 10–12 h. For the evening period, samples were taken with pumping start times between 16:30–21:00 PST (2.5 h before sunset–3.9 h after sunset, mean ± SD relative to sunset 1.4 ± 1.3 h, with 86 % of samples taken after sunset). This wide sampling window was used to capture the potential gradient along the transition from light to dark, which was treated as a linear rather than discrete effect in the analysis (see below). For the day period, samples were taken from the same inflorescence earlier in the same day (start times 12:50–13:50 PST, 0.8–2.0 h after solar noon). Each plant was used on one date only. Dynamic headspace samples of floral volatiles were taken by enclosing inflorescences in 19 x 10 cm nylon-6 oven bags (Reynolds, USA). Volatiles were allowed to equilibrate for 30 min at 22–32 °C (day) or 20–26 °C (evening) and pumped for 30 min through a scent trap using a pump (Supelco PAS-500, Spectrex, Redwood City, California, USA) set to a pre-trap flow rate of 200 mL/min. Ambient controls (*n* = 19) were taken from an empty oven bag sampled for the same duration to identify contaminants (see below). Samples were stored in capped glass vials at -20 °C until analysis.

#### GC-MS Analysis

Floral scent composition (the identity and emission rate of each volatile in the overall scent blend) was characterized and quantified by thermal desorption gas chromatography-mass spectrometry (TD-GC-MS). We employed an Agilent 6890N GC (Agilent Technologies, Palo Alto, California, USA), with a 30 m × 0.25 mm internal diameter x 0.25 μm film thickness HP-5ms column (Agilent). The flow of helium carrier gas was 1 mL/min. Scent traps were placed in the sample tube of a Markes UNITY 2 thermal desorption device, purged with helium for 1 min, heated to 200°C for 5 min while re-trapping on Tenax adsorbent at 25 °C, and desorbed at 200°C for 3 min. After a 2 min hold at 40 °C, the temperature of the GC oven was ramped to 210 °C at 10 °C/min, then to 275 °C at 30 °C/min and held for 2 min. A coupled Agilent 5973N MSD mass spectrometer was operated in electron-impact ionization mode at 70 eV and scanned in the range 50–500 *m/z* at 3 s^-1^.

Peak deconvolution, integration, and tentative compound identification were performed in the Automated Mass Spectral Deconvolution and Identification System (AMDIS) using the NIST 2017 mass spectral library. Components were included if they had mass spectral match scores greater than 75%, had maximum abundances across samples greater than 120,000 counts (6.6% of the median sample), and occurred in more than one sample. After calibration with a C_7_-C_30_ alkane ladder, compound identities were verified by comparing retention indices (RI) with those given in the NIST library. Volatile emission rates were calculated within each compound class from peak integrations by calibration across 4 orders of magnitude with 7 authentic standards ((Z)-hex-3-en-1-ol, α-pinene, indole, linalool, β-caryophyllene, benzaldehyde dimethyl acetate, (E,E)-farnesol) in hexane applied to scent traps. Compounds in floral samples that did not exceed the amounts in ambient controls or GC blanks were considered contaminants (using t-tests with alpha adjusted by the false discovery rate method) and excluded from analyses. Based on the PTR-MS data, oct-1-en-3-ol and (Z)-hex-3-en-1-ol were likely induced by handling the inflorescences because both sharply decreased in the first two hours after bagging. Both compounds can be induced by mechanical damage (Ozawa et al., 2000; Leitner et al., 2005; Kigathi et al., 2009; Boggia et al., 2015). We excluded (Z)-hex-3-en-1-ol from GC-MS analyses because its emissions remained low for days after the initial bagging, but because oct-1-en-3-ol resurged consistently at night (**Supplementary Figure S2**), likely indicating floral emission, we included it in analyses. Emission rates were standardized by the number of open flowers.

#### Statistical Analyses of Scent Composition

The total scent emissions per flower during the evening sampling period were compared between species with a Mann-Whitney test. To identify volatiles that differed between the two species and between times of day, we employed canonical analysis of principal coordinates (CAP; Anderson and Willis, 2003; Campbell et al., 2016) with Bray-Curtis dissimilarities, as implemented in the function *capscale* from the R package *vegan* (R Core Team, 2018; Oksanen et al., 2019). This constrained ordination method is suited to discover multivariate patterns among predefined predictors, in this case, species, time relative to sunset (as a continuous variable because sampling windows were wide), and their interaction. We used a permutation test (*anova.cca*) to test each term of the full model sequentially and determine whether there was a significant interaction after accounting for the main effects. For visualization and to improve interpretation of the time axis, CAP was repeated within each species with time of day as the constraining variable. The CAP method constructs metric multidimensional scaling (MDS) axes to summarize variation that is not explained by the predictors. Volatile emission rates were square-root transformed to reduce skew before analysis.

### Scent Analysis in Real Time by PTR-MS

#### Advantages of Real-Time Sampling

To identify temporal patterns of scent emissions and pollinator activity, most studies have compared scent (all volatiles and their emission rates) and pollinator activity during two discrete daily sampling periods (e.g. Prieto-Benítez et al., 2015). More intensive sampling has yielded qualitative comparisons between selected scent compounds at 1 h resolution and pollinator visitation rates in three daily periods (Dötterl et al., 2012b), and between overall scent intensity at 10 min resolution and a time range of pollinator visits (Dötterl et al., 2012a). To make fine-scale comparisons that quantify scent-pollinator overlap, we take advantage of proton transfer reaction mass spectrometry (PTR-MS) to generate continuous measurements of volatile emissions for multiple days, rather than the average emissions across a sampling period generated by trapping followed by GC-MS. We then compare those time courses with information on timing of pollinator visits at the scale of quarter hours using an overlap statistic to quantify the degree of synchrony. Prior studies with PTR-MS have revealed the daily emission profiles of individual volatiles (Abel et al., 2009), and overlap between thermogenesis and scent signals (Marotz-Clausen et al., 2018), but have not previously been paired with fine scale information on timing of pollinator visits.

Proton transfer reaction time-of-flight mass spectrometry (PTR-MS) allows extremely sensitive, real time quantitation of plant volatile emissions by using hydronium ions for chemical ionization (Lindinger et al., 1998; Jordan et al., 2009). Through direct ionization of the sample gas, PTR-MS can measure small molecules that are not efficiently trapped on adsorbents. Identification of individual components of complex mixtures with PTR-MS is difficult due to fragmentation and overlap of ions at unit mass resolution, but the technique has been used successfully on complex biological samples when paired with GC-MS to positively identify the volatiles expected in the mixture (Eugenio et al., 2007; Cappellin et al., 2012; Masi et al., 2015; Schuhfried et al., 2017). Identifications can be made for ions not found in the GC-MS spectra from standards reported in the literature.

#### PTR-MS Experiment

Emission rates of volatiles are often highly sensitive to the environment (Farré-Armengol et al., 2014; Burkle and Runyon, 2016; Campbell et al., 2019) and thus could differ between the growth chamber and field sites where the moths were studied. To minimize these variations, we sampled floral volatiles for 2–4 days with PTR-MS under environmental conditions similar to the sites where pollinator observations were conducted. Unlike emission rates, timings of volatile emissions are known to be driven by either direct light cues or the circadian clock calibrated by light cues and are not expected to differ relative to those light cues (Fenske et al., 2018). We lined up temporal patterns of volatiles to those that occur under field conditions by expressing time courses relative to the time of sunset or the light-to-dark transition in the photochamber and using light conditions (intensity and photoperiod) and temperature conditions typical of the field. The remaining differences between the field and the photochamber were that temperature was kept constant to observe changes in emission rates not driven directly by heating, and the light transitions were abrupt rather than gradual so that volatiles that respond to light could be distinguished from those with slower regulation. The detailed methods for sampling, data processing, verification with reference standards, and identification are reported in **Supplementary Methods S1**.

#### Statistical Analysis of Volatile Time Courses

To visualize patterns of multivariate change in scent through time and between the species, we performed a principal components analysis of the PTR-MS ion time series with maxima over 0.001 counts·s^-1^·flower^-1^ for all plants at all time points (van Ruth and de Visser, 2015). All ions that met this criterion were analyzed, including those not identified by GC-MS or comparison to reference spectra. Time points were connected with lines to show the progression of each plant through scent space over multiple days. To identify volatiles with similar patterns of emission over time, we constructed WPGMA hierarchical clusterings of Pearson distances (Liao, 2005) among ion time series (with each ion signal scaled to its maximum). The resulting clustering of volatiles, visualized in a clustered heatmap, reflects similarity in both temporal patterns and presence or absence in each species.

To model the temporal peaks of individual volatile emissions, we fit Weibull functions to each ion time series for each plant and each day using the R package *cardidates* (Rolinski et al., 2007). These functions allow different slopes in the rising and falling periods, and different baseline levels before and after the peak. From these fits we extracted the times of the beginning of exponential increase (0.5% of the modelled peak area), maximum, and end of exponential decrease (99.5% of the modelled peak area). For each species, we calculated the median time of maxima for each ion across days and plants.

To quantify the degree of scent-pollinator synchrony in *S. kaalae*, we compared the 24 h distributions of *P. brevipalpis* visits across all dates to both a) the modelled times of maximum emission (from the fitted Weibull function) aggregated across all PTR-MS ions, days, and plants (which provides a single metric of synchronization between pollination and the timing of peaks across all scent compounds) and b) the actual time courses of emissions for each PTR-MS ion across days and plants (which shows which volatiles are the most or least synchronized with pollination). After aligning the sunset time to the dark transition in the growth chamber, we placed times of moth visits into bins that were 16 min in duration, centered on the 4-min sampling blocks for each plant. We normalized each distribution to have an area of one, and then calculated the areal overlap between the two distributions (defined as the integral of the minimum of the two distributions; Miller-Rushing et al., 2010). This statistic is affected by the position of the two distributions relative to each other, and the match in their width. We define the null expectation as the overlap between the moth visit distribution and a flat line, where the flat line represents either a) a uniform distribution of times of maxima or b) a hypothetical volatile holding a constant emission rate throughout the day.

### Compounds Attractive to Moths

Selection for overlap between emission of a specific compound and moth visitation might be more likely if the compound is one that moths respond to behaviorally. The behavioral responses of *Pseudoschrankia brevipalpis* to individual floral volatiles are unknown, so we surveyed the literature for information on the detectability (search terms: moth + {antenna, EAD, EAG}) and attractiveness (search terms: moth + {attraction, behavior}) of the volatiles produced by *Schiedea* inflorescences. Electroantennographic detection (EAD) studies were used to determine whether a compound can be detected by moth antennae. Evidence of moth attraction is presented from behavioral tests. In these studies, the volatile was considered attractive if it induced more interactions than the control. Volatiles were applied to either an open trap with a scent emitter, a scent emitter within a wind tunnel, or a flower spiked with additional scent. From the literature, we recorded the number of moth species, their families, and the apparatus used (**Supplementary Table S2**).

## Results

### Species Differences in Floral Scent

Using GC-MS we detected 32 floral volatiles produced by *S. kaalae* and 36 produced by *S. hookeri*, for a total of 40 volatiles present in > 20% of samples of either species. These included 19 aliphatics, 7 benzenoids, 5 irregular terpenes, and 9 monoterpenes (**Table 1**). Of the 40 compounds, 28 were produced by both species. The literature survey of electrophysiological and behavioral studies in other moth species showed 9 are EAD-active (with no behavioral data available), 12 are EAD-active and attractive, one is not attractive, and no data are available for the others (**Supplementary Table S2**). Including rarer volatiles and excluding two putative wound volatiles, a total of 74 volatiles were detected and used for analysis.

**Table 1:** Evening floral volatile emissions from *Schiedea kaalae* and *S. hookeri* detected by GC-MS in > 20% of samples of either species (40 of 76 compounds, *n* = 32 plants for each species).

Class	RI ^1^	Mean match score	CAS ^2^	Name	Proportion of evening samples^3^		Mean nonzero emission rate (ng/flower/hr)		Mean emission rate (ng/flower/hr)		Mean relative emission rate ^4^	
					*S.kaalae*	*S.hookeri*	*S.kaalae*	*S.hookeri*	*S.kaalae*	*S.hookeri*	*S.kaalae*	*S.hookeri*
Aliphatic	796	87%	4440-65-7	(E)-hex-3-enal	0%	50%		0.56		0.28		3.4%
	797	94%	66-25-1	hexanal	**100%**	**100%**	0.56	0.80	0.56	0.80	3.4%	9.5%
	830	90%	96-04-8	heptane-2,3-dione	8%	**86%**	0.05	0.54	0.00	0.46	0.0%	5.6%
	840	91%	6728-26-3	(E)-hex-2-enal	0%	**81%**		0.43		0.35		4.2%
	848	94%	928-96-1	(Z)-hex-3-en-1-ol	21%	**89%**	0.24	6.64			excl.	excl.
	855	78%	7642-10-6	hept-3-ene	5%	22%	0.24	0.26	0.01	0.06	0.1%	0.7%
	855	84%	4412-91-3	furan-3-ylmethanol	8%	25%	0.10	0.95	0.01	0.24	0.0%	2.8%
	855	89%	2415-72-7	propylcyclopropane	32%	25%	0.08	0.33	0.03	0.08	0.2%	1.0%
	882	84%	2216-34-4	4-methyloctane	21%	3%	0.03	0.01	0.01	0.00	0.0%	0.0%
	901	92%	13129-23-2	methyl furan-3-carboxylate	0%	33%		0.03		0.01		0.1%
	905	92%	3008-40-0	cyclopentane-1,2-dione	21%	22%	0.78	1.85	0.16	0.41	1.0%	4.9%
	933	83%	18829-55-5	hept-2-enal	37%	0%	0.06		0.02		0.1%	
	949	79%	26456-76-8	3,5,5-trimethylhex-2-ene	5%	47%	0.05	0.40	0.00	0.19	0.0%	2.3%
	960	92%	3391-86-4	oct-1-en-3-ol	**100%**	**100%**	3.17	3.38	3.17	3.38	19.3%	40.6%
	961	92%	106-68-3	octan-3-one	**89%**	**97%**	0.49	0.72	0.44	0.70	2.7%	8.4%
	971	84%	111-13-7	octan-2-one	21%	0%	0.04		0.01		0.0%	
	981	92%	72237-36-6	hex-4-enyl acetate	0%	25%		0.35		0.09		1.1%
	1152	87%	53398-84-8	[(E)-hex-3-enyl] butanoate	0%	28%		0.29		0.08		1.0%
	1347	90%	31501-11-8	[(Z)-hex-3-enyl] hexanoate	0%	28%		0.27		0.07		0.9%
Benzenoid	896	91%	100-66-3	anisole	3%	39%	0.01	0.02	0.00	0.01	0.0%	0.1%
	937	95%	100-52-7	benzaldehyde	**100%**	**100%**	0.07	0.30	0.07	0.30	0.5%	3.7%
	1017	92%	122-78-1	2-phenylacetaldehyde	**95%**	50%	0.29	0.01	0.27	0.01	1.7%	0.1%
	1179			unknown benzenoid ^5^	11%	**64%**	0.02	0.42	0.00	0.27	0.0%	3.2%
	1198	86%	103-70-8	N-phenylformamide	0%	25%		0.09		0.02		0.3%
	1268	92%	120-72-9	indole	13%	**89%**	0.02	0.16	0.00	0.15	0.0%	1.8%
	1316	94%	134-20-3	methyl 2-aminobenzoate	5%	**61%**	0.00	0.14	0.00	0.08	0.0%	1.0%
Irregular terpene	1086	82%	19945-61-0	(3E)-4,8-dimethylnona-1,3,7-triene	3%	**64%**	0.05	0.15	0.00	0.09	0.0%	1.1%
	1115	90%	1125-21-9	4-oxoisophorone	**100%**	3%	0.13	0.00	0.13	0.00	0.8%	0.0%
	1120	87%	28564-83-2	3,5-dihydroxy-6-methyl-2,3-dihydropyran-4-one	16%	22%	0.14	0.13	0.02	0.03	0.1%	0.3%
	1139	87%	20547-99-3	2,2,6-trimethylcyclohexane-1,4-dione	**84%**	0%	0.06		0.05		0.3%	
	1322	81%	141891-14-7	4-hydroxy-2,6,6-trimethyl-3-oxocyclohexene-1-carbaldehyde	**71%**	3%	0.04	0.04	0.03	0.00	0.2%	0.0%
Mono-terpene	964	84%	123-35-3	β-myrcene	47%	3%	0.02	0.02	0.01	0.00	0.1%	0.0%
	978	90%	99-83-2	α-phellandrene	**76%**	42%	0.46	0.04	0.35	0.02	2.2%	0.2%
	997	89%	99-87-6	p-cymene	**55%**	22%	0.12	0.06	0.07	0.01	0.4%	0.2%
	1013	85%	3779-61-1	(E)-β-ocimene	26%	0%	0.02		0.00		0.0%	
	1061	94%	5989-33-3	linalool oxide (furanoid)	**100%**	22%	2.13	0.08	2.13	0.02	13.0%	0.2%
	1071	82%	78-70-6	linalool	34%	47%	0.11	0.05	0.04	0.03	0.2%	0.3%
	1087	93%	33933-72-1	linalool oxide (pyranoid) ketone	**100%**	**75%**	4.82	0.11	4.82	0.08	29.4%	1.0%
	1152	93%	39028-58-5	linalool oxide (pyranoid)	**100%**	22%	3.98	0.10	3.98	0.02	24.2%	0.3%
	1245	78%	EPA-7965	epoxy-linalooloxide	39%	0%	0.04		0.02		0.1%	

Evidence of EAD (electroantennographic detection) responses or attraction of moths for these compounds is presented in **Supplementary Table S2**.

^1^ ****Kovats retention index (RI). ^2^ CAS registry number or NIST library number. ^3^ Percentage of all evening samples in which the compound was detected, with entries > 50% in bold. ^4^ The mean of emission rates scaled to 100%. ^5^ Fragment ions relative to m/z 91 (100%): 65 (20%), 119 (19%), 162 (11%), 92 (9%), 63 (8%), 89 (6%), 51 (5%). NIST MS Search “Substructure Information” analysis indicates molecular mass of 162, probable disubstituted phenyl with a carbonyl group.

**Figure 1:**
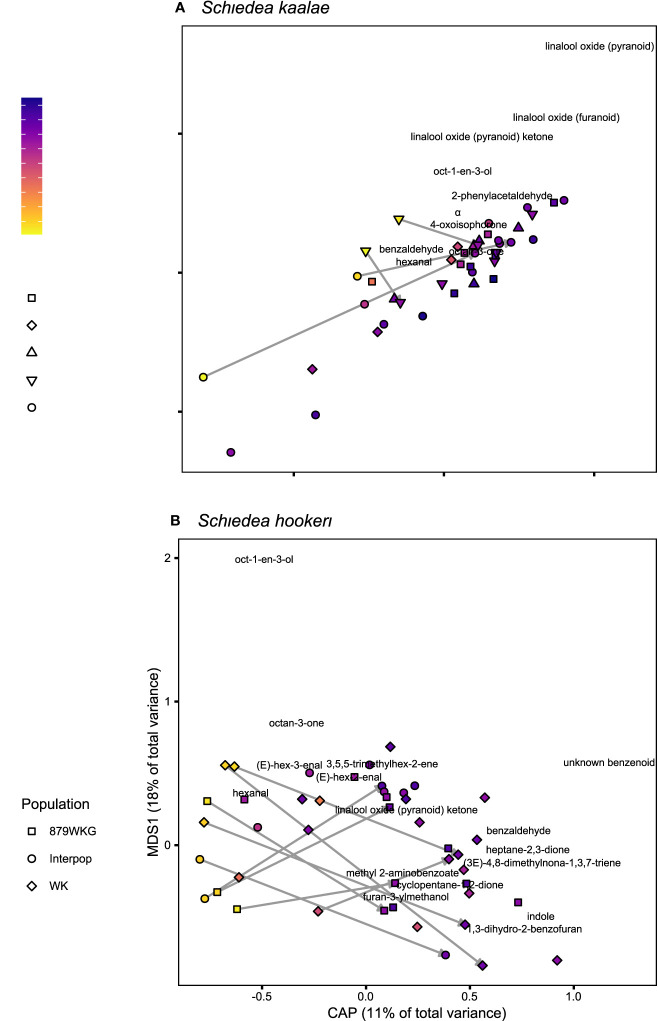
Floral scent composition determined by GC-MS of **(A)** *Schiedea kaalae* and **(B)** *S. hookeri* plants sampled at different times of day, visualized by canonical analyses of principal coordinates (CAP). The CAP axis shows scent variation explained by time of day (evening on the right), and the first multidimensional scaling (MDS) axis shows additional unconstrained scent variation. The shape of the points indicates the source population number (collection locations in **Supplementary Table S1**) or a plant from a cross between populations (“Interpop”). Color indicates the time of collection, with zero indicating sunset, positive values indicating time after sunset, and negative values indicating time before sunset. Arrows connect samples of the same inflorescence during the day and following night. The names of volatiles are positioned by their CAP and MDS scores and labelled if they are > 0.05 units from the origin.

*Schiedea kaalae* produced more total scent per flower than *S. hookeri* in the evening in the GC-MS measurements (median ± median absolute deviation 23 ± 12 ng·flower^-1^·h^-1^ compared to 5.0 ± 3.9 ng·flower^-1^·h^-1^ for *S. hookeri*, Mann-Whitney test, *U* = 179, *P* < 10^-10^). Major components of the scent blends differed (CAP species effect, **Table 2A**). For *S. kaalae* in the evening, three cyclic linalool oxides (the pyranoid oxide ketone, pyranoid oxide, and furanoid oxide) made up 67% of the average scent blend, followed by five volatiles each making up more than 1.5% of the blend: oct-1-en-3-ol, hexanal, octan-3-one, α-phellandrene, and 2-phenylacetaldehyde (**Table 1**, **Figure 1A**). The evening blend was more complex for *S. hookeri* than *S. kaalae* (Shannon diversity index of 2.1 ± 0.3 [mean ± SD] versus 1.6 ± 0.2 for *S. kaalae*), and composed of oct-1-en-3-ol (41%), followed by 11 volatiles each making up 1.5–10% of the blend: hexanal, octan-3-one, heptane-2,3-dione, cyclopentane-1,2-dione, two hexenal isomers, benzaldehyde, an unknown benzenoid, furan-3-ylmethanol, 3,5,5-trimethylhex-2-ene, and indole (**Table 1**, **Figure 1B**). The first CAP axis that separated the floral scents of the species reflects these major differences (**Table 2B**).

**Table 2:** Canonical analysis of principal coordinates (CAP) of the effects of species (*Schiedea kaalae* or *S. hookeri*) and time of day on floral scent composition. (A) ANOVA-like permutation test (*n* = 99999 iterations) of each term. (B) Compound scores on the first CAP axis, which discriminated between the species. Absolute scores ≥ 0.02 are included. Negative values indicate compounds associated with *S. hookeri*, and positive values indicate compounds associated with *S. kaalae*.

(A) Test of CAP model				
	df	SS	F	P
**Species**	1	6.74	52.7	0.00001
**Time**	1	0.61	4.8	0.00146
**Species : Time**	1	0.37	2.9	0.02012
**Residual**	72	9.20		
**(B) Compounds separating species**				
**Name**			**CAP1 Score**	
			*S. hookeri*	
unknown benzenoid			-0.09	
indole			-0.08	
(E)-hex-2-enal			-0.07	
(E)-hex-3-enal			-0.06	
methyl 2-aminobenzoate			-0.06	
heptane-2,3-dione			-0.06	
1,3-dihydro-2-benzofuran			-0.06	
benzaldehyde			-0.05	
(3E)-4,8-dimethylnona-1,3,7-triene			-0.05	
3,5,5-trimethylhex-2-ene			-0.04	
anisole			-0.02	
N-phenylformamide			-0.02	
furan-3-ylmethanol			-0.02	
2,2,6-trimethylcyclohexane-1,4-dione			0.06	
oct-1-en-3-ol			0.06	
4-oxoisophorone			0.09	
α-phellandrene			0.12	
2-phenylacetaldehyde			0.12	
linalool oxide (furanoid)			0.43	
linalool oxide (pyranoid)			0.59	
linalool oxide (pyranoid) ketone			0.62	
			*S. kaalae*	

**Figure 2:**
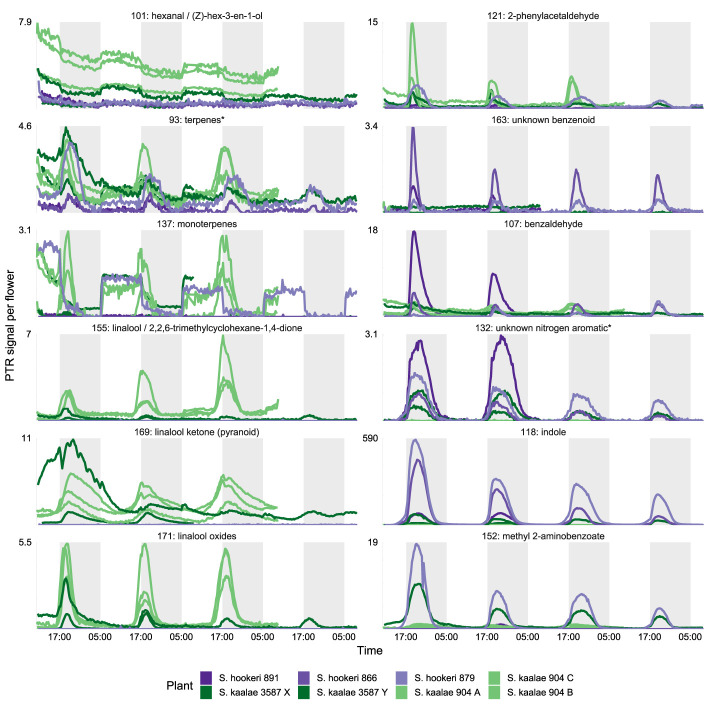
PTR-MS signals per flower (arbitrary units) for floral volatile emissions from five *Schiedea kaalae* plants (green shades) and three *S. hookeri* plants (purple shades) across 2–4 d. Periods of darkness in the growth chamber are indicated by darker gray shading. Plants are named with their population number and a letter (collection locations in **Supplementary Table S1**), and colored by population. Panels present those PTR-MS ion signals (*m/z* value given in the label) that correspond to molecular or fragment ions of volatiles identified by GC-MS in evening scent emissions. Scales vary according to the maximum signal per flower, displayed next to each panel.

**Figure 3:**
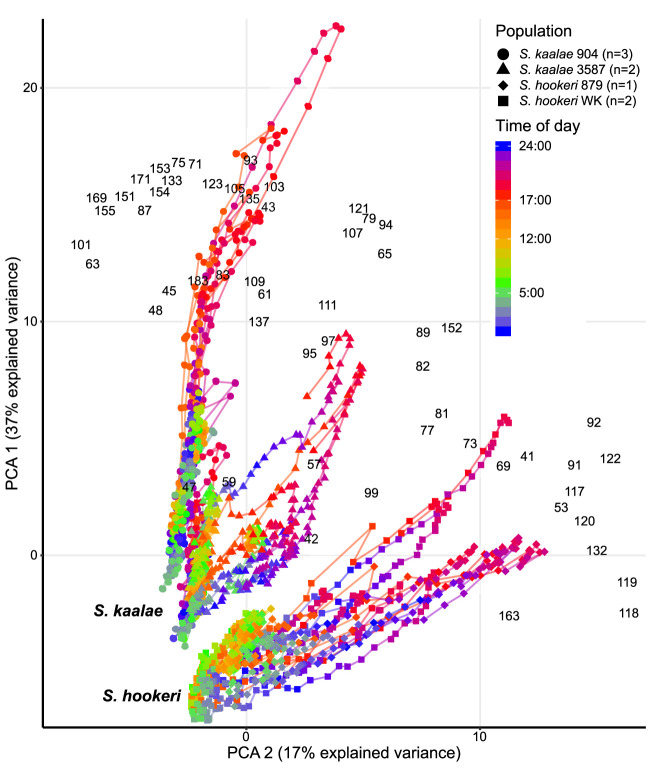
Daily patterns of floral scent in the same five *Schiedea kaalae* plants and three *S. hookeri* plants as in Figure 2 mapped by principal components analysis (PCA) of PTR-MS ion signals with maxima over 0.001 counts·s^-1^·flower^-1^, including unidentified ions. The first and second principal components are shown on the vertical and horizontal axes. Loadings for each ion are indicated by the black *m/z numbers* (**Supplementary Table S3**). Lines connect adjacent time points for each plant. Time of day is represented by different colors on the line, with transitions from dark to light at 5:00 (cyan-green) and light to dark at 17:00 (orange-red) marked on the scale. The source population (904, 3587, 879, WK) is indicated by the shape of the points (collection locations in **Supplementary Table S1**). Each plant was sampled for 2–4 d.

These differences in evening scent between the two species were supported by PTR-MS measurements (**Figure 2**). The two species produced distinct scent blends at all times of day (principal components analysis of ions in the PTR-MS spectrum across all timepoints, **Figure 3**). The scent compositions of the two species were most distinct from each other during the evening (**Figure 3**) and this was verified by the full CAP analysis of GC-MS volatile compositions (**Table 2A**, ordination not shown). Individuals from the two *S. kaalae* populations differed from each other in their evening scent composition (**Figure 3**), primarily by the emission of indole by the two plants from Puʻumaialau (Takeuchi 3587) which was absent in the three plants from Pahole Gulch (Weller & Sakai 904; both in the Waiʻanae range, **Figure 2**, **Supplementary Table S1**).

### Daily Patterns in Floral Scent

#### Comparisons Between Day and Night Using GC-MS

In both *Schiedea kaalae* and *S. hookeri*, total floral scent emissions increased and scent composition changed markedly in the evening. Median evening scent emissions measured by GC-MS for *S. kaalae* were 1.5 times higher than daytime emissions and 1.8 times higher for *S. hookeri*. Scent composition varied by species, time of day, and their interaction (full canonical analysis of principal coordinates, **Table 2A**). The scent composition of individual plants changed between the day and evening within both species (time effects in separate CAP analyses: F_1,38_ = 6.17, P = 0.0001 for *S. hookeri* and F_1,34_ = 3.11, P = 0.0024 for *S. kaalae*). For *S. kaalae*, the volatiles with the highest evening loadings were linalool oxide (pyranoid), linalool oxide (furanoid), and 2-phenylacetaldehyde (**Figure 1A**), all of which are EAD-active in moths (**Supplementary Table S2**). In *S. hookeri*, volatiles with the highest evening loadings were the unknown benzenoid, 1-3-dihydro-2-benzofuran, and indole (**Figure 1B**; indole attracts hawkmoths, **Supplementary Table S2**).

#### Fine Scale Timing Using PTR-MS

**Figure 4:**
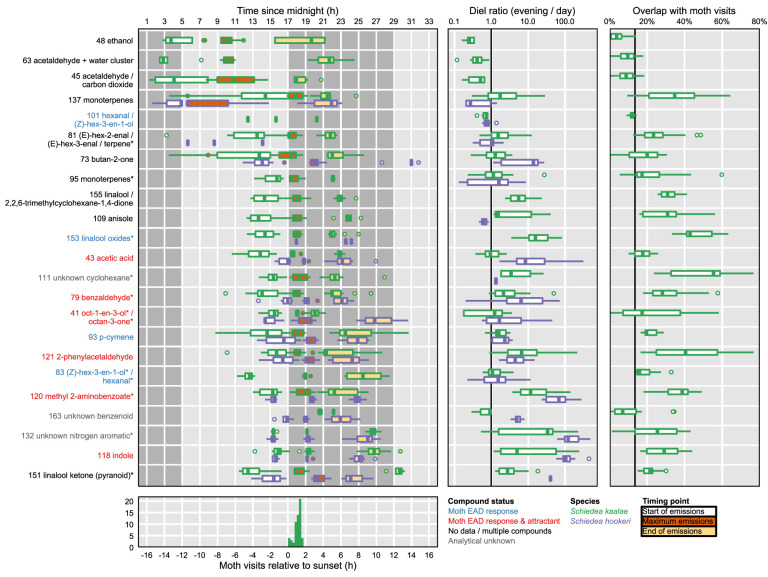
Moth foraging activity and summaries of temporal peaks of floral volatile emissions of *Schiedea kaalae* (green) and *S. hookeri* (purple). Boxplots contain the median, first and third quartiles, range, and outliers (beyond 1.5 times the interquartile range from the first or third quartile). *Left:* The timing of volatiles emissions. Tentative identifications for each ion are given after their protonated *m/z* value. Fragment ions are indicated by an asterisk by the name. One PTR-MS ion per compound is shown (**Supplementary Table S3**), for ions with maxima over 0.001 counts·s^-1^·flower^-1^ for all plants at all time points. For each ion and species, three boxplots summarize (across all plants and days) the start (white), maximum (maroon), and end (light orange) of emissions. Timing points were inferred by fitting Weibull functions to ion signals and trimming to 99% of the fitted peak area. Ions are arranged vertically by the mean starting time relative to the dark transition in the growth chamber. Light and dark periods in the growth chamber are indicated by background shading. The dark transition in the growth chamber was approximately coincident with the ambient greenhouse sunset time. Ion labels are colored by whether they elicit a moth antennal EAD response (blue), elicit an EAD response and attraction (red), are not reported in the literature (black, labelled ‘no data’), contain signals from multiple compounds (black), or are analytical unknowns (gray, all references in (**Supplementary Table S2**) except acetic acid, Knight et al., 2011). *Middle:* The magnitude of daily changes in emission of floral volatiles. Boxplots show the diel ratio in emissions (evening/day) for evening (19:00–20:00 PST, 2–3 h after dark) and day (12:00–13:00 PST, 5–4 h before dark) for each plant and date. A ratio > 1 (right of vertical bar) indicates that emissions increased in the evening. *Right*: The overlap of *S. kaalae* volatile emissions with moth activity. Boxplots show the areal overlap value between two curves: the time course of each volatile relative to dark, and the distribution of *P. brevipalpis* visit times relative to sunset. Overlap values vary among plants and days. The vertical bar indicates the null overlap expectation for a hypothetical volatile that does not change in emission over the course of a 24 h period (14% overlap). *Bottom:* Visits of *Pseudoschrankia brevipalpis* to *S. kaalae* flowers relative to sunset at the field site over four dates.

The floral scents of both species intensified in the evening in the PTR-MS measurements (**Supplementary Table S3**, **Figure 4**) as they did with GC-MS. This daily modulation was driven by pulses of individual volatiles from diverse biochemical pathways with periodicity of approximately 24 h (**Supplementary Figure S2**). Each volatile had a distinctly-shaped time course (**Figure 2**) but the times of maximum emission among the evening volatiles fell within a 4 h period (**Figure 4**). The volatile emission patterns formed three main groups based on their starting times relative to the light and dark transitions (**Supplementary Table S3**). Morning volatiles, such as acetaldehyde (m/z 45), started to rise from their baseline emission rates when plants are exposed to light, plateaued near their maximum within 1 h, began to fall at dark, and returned to baseline 1–5 h after dark. Afternoon volatiles, such as linalool ketone (pyranoid) (m/z 169), rose 0–6 h before dark, peaked 0–2.5 h after dark, and returned to baseline 4–10 h after dark. Some of the afternoon volatiles that started rising slowly in the afternoon showed an inflection point at the dark transition and began rising more quickly (**Figure 2**, e.g. indole). Dark volatiles, such as benzaldehyde (m/z 107), rose at dark, peaked 1–3 h after dark, and returned to baseline 3–8 h after dark.

Both species started emitting more volatiles in the afternoon or after dark than in the morning (**Supplementary Table S3**). Production of all of the known moth attractants started in the afternoon or after dark (**Figure 4**). Daytime emission rates for many evening-peaking volatiles were generally very low, on the order of tens to hundreds of times less than emission rates in the evening (**Figure 4**, **Supplementary Table S3**), although some daily changes were more subtle [e.g. linalool oxide (pyranoid) ketone in *S. kaalae*; **Figure 2**]. The magnitude of the diel ratio (the emission rate 2–3 h after dark relative to the rate 5–6 h before dark) varied between species for the same volatile (**Figure 4**, **Supplementary Table S3**); for example, *S. hookeri* showed more extreme increases at night than *S. kaalae* in methyl 2-aminobenzoate, indole, and the unknown nitrogen aromatic and unknown benzenoid. The temporal patterns were consistent across days, plants, and in some cases between species, although the volatile emissions of *S. hookeri* often started and peaked later compared to the same compound in *S. kaalae* (**Figure 4**). In some plants, maximum emissions of some volatiles varied over consecutive days and generally decreased over time, perhaps due to aging of the inflorescence (**Figure 2**, **Supplementary Figure S2**).

### Overlap With Moth Visitation

*Pseudoschrankia brevipalpis* visited *S. kaalae* in ʻĒkahanui Gulch from 0.2–1.6 h after sunset (mean ± SD 1.1 ± 0.4 h after sunset, *n* = 48). For *S. kaalae*, most volatiles began emission 1–5 h before the first *P. brevipalpis* visit to any flowers, peaked 1.5 h before–1 h after the mean time of moth visits, and returned to baseline 1–4 h after the last visit (times relative to sunset or the dark transition in the growth chamber, **Figure 4**).

The areal overlap between the time of moth visitation to *S. kaalae* and the times of maxima across PTR-MS ions, days, and plants was 49%, much greater than the null expectation of 14% for a uniform distribution of maxima, given these moth observations. The median time of ion maxima was 1.6 h after dark for *S. kaalae* and 2.4 h after dark for *S. hookeri*. The time courses of individual *S. kaalae* volatiles varied in their degree of overlap with moth visitation (**Figure 4**), with an unknown cyclohexane, the linalool oxides, 2-phenylacetaldehyde, and methyl 2-aminobenzoate having the highest overlap (both 2-phenylacetaldehyde and methyl 2-aminobenzoate are moth attractants; **Supplementary Table S2**). The mean overlap for the individual time courses of *S. kaalae* volatiles and moth visits was 25 ± 16%, 25 ± 15% for EAD-active volatiles, and 30 ± 16% for moth attractants that were EAD-active (mean ± SD), compared to a null expectation of 14% overlap for volatiles emitted at a constant rate. Volatiles that rose in the morning and peaked during the day (such as acetaldehyde) had low overlap with moth visitation, and of this group only the green leaf volatile (Z)-hex-3-en-1-ol (m/z 101, PTR-MS ion signal shared with hexanal) was an attractant, for moths that feed on leaves (**Supplementary Table S2**). The degree of overlap also varied across nights and plants, driven primarily by variation in the diel ratio and secondarily by changes in the timing of the maximum (**Figure 4**).

## Discussion

In two *Schiedea* species pollinated by the same moth, the timing of emission of floral volatiles was more similar than the identity of the major compounds released by those species in the evening. The floral scents produced by *S. kaalae* and *S. hookeri* were notable for the biochemical diversity of compounds that oscillate between day and night. The timings of peak pollinator activity for *S. kaalae* and of peak emissions of known moth attractants was similar, although volatile emissions started prior to pollinator activity and continued after cessation of pollinator activity.

### Moth Attractants

Many volatiles that peak in the evening in *S. kaalae* and *S. hookeri* are typical benzenoid, oxygenated terpene, and nitrogen-containing floral attractants of crepuscular noctuid and sphingid moths (**Supplementary Table S2**), such as those found in the nocturnal floral emissions of moth-pollinated orchids (Kaiser, 1993), *Nicotiana* (Loughrin et al., 1991), *Petunia* (Hoballah et al., 2005), and other diverse taxa (Knudsen and Tollsten, 1993; Dobson et al., 1997; Miyake et al., 1998). In other studies, the hawkmoth *Hyles lineata* shows antennal responses to many volatiles emitted in the evening by the two *Schiedea* species (**Supplementary Table S2**).

The potential attractive role of these nocturnally-emitted compounds in *Schiedea* is highlighted by their increase in production with evolutionary transitions to moth pollination in several other genera. In *Clarkia*, production of linalool and linalool oxides (the pyranoid and furanoid forms produced by *S. kaalae*) evolved in a transition from bee to nocturnal moth pollination (Raguso and Pichersky, 1995). In *Ipomopsis*, indole (in our study produced primarily by *S. hookeri*) attracts hawkmoths to *I. tenuituba* but is not emitted by its hummingbird-pollinated sister species *I. aggregata* (Bischoff et al., 2015). *Nicotiana bonariensis* produces the apocarotenoid 4-oxoisophorone and its variant 2,2,6-trimethylcyclohexane-1,4-dione (both produced by *S. kaalae*) from flowers that open at dusk and are pollinated by small crepuscular moths (Noctuidae) rather than the hawkmoths and hummingbirds attracted to close relatives of *N. bonariensis* that lack these compounds (Raguso et al., 2003; Clarkson et al., 2004; Kaczorowski et al., 2005). None of the evening-peaking volatiles in *Schiedea hookeri* and *S. kaalae* were present in the wind-pollinated *Schiedea* species (*S. globosa* and *S. kealiae*, Jürgens et al., 2012) that *P. brevipalpis* largely avoided in field choice tests (Weller et al., 2017).

### Species Differences in Floral Scent

In this study, *S. kaalae* and *S. hookeri* share a sole pollinator in an area of sympatry, but have different evolutionary histories, leading us to predict distinct floral volatile compositions. In sympatric species from different lineages of sexually-deceptive and oil-secreting orchids, similar selection pressures imposed by the same pollinator have driven convergence in overall floral scent, or in the subset of compounds that have antennal activity (Cortis et al., 2009; Gögler et al., 2009; Nunes et al., 2017). We found instead that the evening floral scents of the two *Schiedea* species pollinated by *P. brevipalpis* differ qualitatively in composition. Scent differences between the species are more accentuated during the evening than during the day, echoing the same pattern found in nine *Nicotiana* species, some of which are nocturnally pollinated by hawkmoths (Raguso et al., 2003). The overall composition and major compounds of each species are unique: *Schiedea kaalae* produces a set of three linalool oxides and 2-phenylacetaldehyde, which are produced in relatively minute amounts by *S. hookeri*, and *S. hookeri* uniquely produces an unknown benzenoid and heptane-2,3-dione (**Table 1**). These qualitative differences could result from the evolutionary history of *S. hookeri*, which is in a clade of wind-pollinated species (*Schiedea* sect. *Schiedea*) and may represent a reversal to moth pollination from ancestral wind pollination (the current phylogenetic hypothesis does not fully resolve the direction of this shift, Willyard et al., 2011). However, both *S. kaalae* and *S. hookeri* produce the moth attractant benzaldehyde (Hoballah et al., 2005) and the insect attractant oct-1-en-3-ol (Hall et al., 1984), and *S. hookeri* emits the moth attractants indole (Bischoff et al., 2015) and methyl 2-aminobenzoate (Bisch-Knaden et al., 2018) which are emitted at lower rates by *S. kaalae* (**Figure 2**). Experiments that test moth preferences in the field at sites of both species (as in Bischoff et al., 2015) are needed to elucidate whether one critical volatile, a blend of the shared volatiles, or other factors are important for attraction of pollinators. Given the observed differences in scent between these related species that share the same moth species as a pollinator, future community studies should not always assume strict similarity in scent composition across unrelated plant taxa visited by the same pollinator or pollinator guild. Instead, distinct sets of compounds may be perceived by those pollinators.

### Overlap With Moth Visitation

Our work builds on diverse examples of synchrony in floral signals and pollinator activity during the day (Matile and Altenburger, 1988; Kite and Smith, 1997; Dötterl et al., 2012a; Nunes et al., 2016) and night (e.g. Nilsson, 1983; Dötterl et al., 2005; Hoballah et al., 2005; Dötterl et al., 2012b; Steen et al., 2019) and enhances temporal resolution to characterize the overlap of pollinator activity and floral volatile production. In both *Schiedea* species, the emissions of many floral volatiles were restricted to the afternoon and evening hours and in *S. kaalae* peaked within 2 h of the mean time of *P. brevipalpis* visits in the field (**Figure 4**). In *S. kaalae*, the distribution of timings of maximum emissions across all volatiles, days, and plants indicated a good but imperfect temporal match between potential signals and the insect receiver. The volatiles that peak during the day and fall at dark would not be perceived by crepuscular moths after sunset, and their patterns of emission were all consistent with induction by light. The daytime volatiles could be related to photosynthesis (in the bracts of *S. hookeri*) or transpiration, rather than pollinator attraction (as is the case for both the daytime-peaking acetaldehyde and ethanol, its precursor; Graus et al., 2004). The maximum emissions of *S. hookeri* evening volatiles were shifted about 1 h later on average than their counterparts in *S. kaalae*, and many *S. hookeri* volatiles continued to be emitted until the early morning. These differences could stem from alternate temporal selection pressures (perhaps moths visit *S. hookeri* at a later time than they visit *S. kaalae*), or differences in evolutionary history of the plant species.

In *S. kaalae*, many volatile emissions spanned a much broader time range than the period of moth visitation. This could indicate constraints on how fast volatile emissions can be modulated, low ecological costs (e.g. apparency to herbivores) or low energetic costs of volatiles at those times, or a marginal benefit of attracting any moths that may be active at those times. Early initiation of volatile emission (i.e., for the volatiles that rose in the afternoon) could create a long downwind scent plume for long-distance attraction of moths (**Supplementary Table S3**, Cardé and Willis, 2008). Conversely, the volatiles that rise after dark just as moths are beginning to forage could be important for short-distance attraction. The peaks of individual *S. kaalae* evening volatiles differed in their degree of overlap with the distribution of moth visitation (20–55%; **Figure 4**). Known moth attractants, but not EAD-active volatiles, had slightly higher areal overlap in time with moth visits than the mean across all volatiles. This areal overlap statistic captured temporal differences from both early or late shifts in the time course of emissions and differences in peak width (narrow or broad), the two types of differences that are characterized in studies of phenology (Miller-Rushing et al., 2010). These two components were also examined separately by calculating times of maxima and diel ratios. Either type of difference could affect how and when pollinators or other visitors could perceive these volatiles.

Daily regulation of attractants may increase the fitness of plants by reducing energetic costs, and it may also serve to reduce the attraction of plant antagonists that use the same floral cues as pollinators (Baldwin et al., 1997; Nunes et al., 2016). No native florivores or herbivores have been reported on outplanted or natural populations of these or any other *Schiedea* species. Though the fitness costs of emitting the evening volatiles during the day are unknown, the high level of daytime and before-dawn suppression indicates they could be substantial.

Floral scent is a complex trait in both synthesis and perception, and identification of volatiles or suites of volatiles that serve different functional roles (defense, attraction, metabolism) within diverse scent blends is challenging. However, categorizing volatiles by their pattern of temporal regulation (Nielsen et al., 1995; Marotz-Clausen et al., 2018) narrows the set of compounds that potentially influence the behavior of pollinators with constrained windows of activity. Follow-up behavioral studies might be able to test these candidate volatiles to confirm a function. In this case, volatiles could be classified by whether they increased immediately with light (e.g. monoterpenes), increased in the afternoon without a light cue (e.g. pyranoid linalool ketone), or increased after dark (e.g. benzaldehyde; **Figures 2** and **4**). Volatiles could also be ranked by their relative change in emission rate when the pollinator is active vs. not active, and by their overlap with pollinator visitation. Future studies could investigate the proximate causes of regulation of these volatiles (e.g. by the circadian clock, reviewed in Fenske and Imaizumi, 2016), and identify which class is most attractive to pollinators. We predict that the afternoon-rising volatiles are long-range attractants because they would diffuse a great distance by the time moths are active, allowing moths to detect the population. Volatiles that increase after dark may be short-range attractants because they would not establish a long scent plume by the time moths are active.

## Conclusions

Almost all volatiles released from inflorescences of *Schiedea kaalae* and *S. hookeri* displayed strongly time-specific modulations. Most *S. kaalae* volatiles peaked during or several hours after the brief time of evening visitation of *Pseudoschrankia brevipalpis*, a pollinator of both species. This pattern is generally consistent with selection that maximizes the attraction of pollinators by producing volatiles when pollinators are active, but the emission of most evening volatiles extended hours before the period of pollinator activity, when they could be active in long-range attraction. Additionally, some volatiles, perhaps unrelated to pollinator attraction, followed a daytime cycle. The composition of volatiles differed markedly between species, especially in the evening, and yet the timings of peak emissions were similar between the species. Knowing when emissions of each volatile begin, peak, and end will help to focus studies on the ecological functions of volatile compounds based on their temporal overlap with the activity of mutualists and antagonists.

## Data Availability Statement

The datasets presented in this study can be found in online repositories. The names of the repository/repositories and accession number(s) can be found below: https://doi.org/10.7280/D12H4M. The data repository is Dryad.

## Author Contributions

All authors participated in the design of the experiment. DC, RS, and CF provided advice on volatile analysis, and AS and SW provided advice on the study system. AG and RS designed and provided equipment. JP and RS collected and analyzed the data. JP wrote the manuscript, and all authors contributed substantially to revisions.

## Funding

This work was supported by the University of California, Irvine (Graduate Fellowship to JP and CORCL multi-investigator grant to DC, SW, Katrine Whiteson, AS, and Kailen Mooney) and by the National Science Foundation (DEB 1753664 to AS and SW, co-PIs).

## Conflict of Interest

The authors declare that the research was conducted in the absence of any commercial or financial relationships that could be construed as a potential conflict of interest.
