# Supplementary material for: Floral Scent Composition and Fine-Scale Timing in Two Moth-Pollinated Hawaiian Schiedea (Caryophyllaceae)
Source: Front Plant Sci. 2020 Jul 21;11:1116. doi: 10.3389/fpls.2020.01116 (PMC7385411; doi:10.3389/fpls.2020.01116)

## Supplementary Figure S1

PTR-MS spectra of reference standards. Only ions with signals > 2 times the initial zero air reading are included. Scales vary between plots, as compounds with higher vapor pressures (Kim *et al.,* 2016) produced higher total filtered signals. The molecular ion M[H+] of each compound is indicated in parentheses.

### *Reference*

Kim, S., Thiessen, P. A., Bolton, E. E., Chen, J., Fu, G., Gindulyte, A., et al. (2016). PubChem Substance and Compound databases. *Nucleic Acids Res* 44, D1202–D1213. doi:[10.1093/nar/gkv951](https://doi.org/10.1093/nar/gkv951).


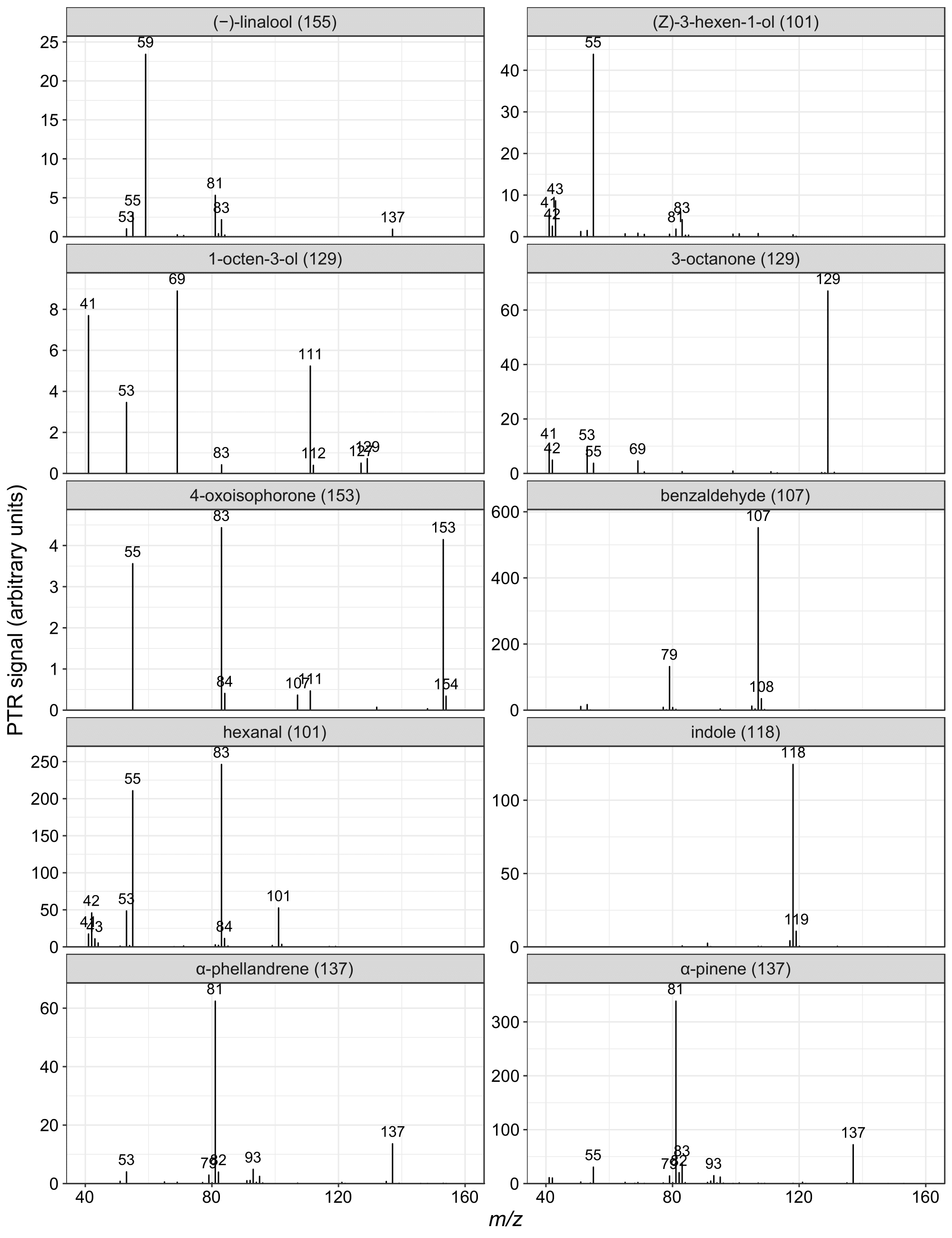

Supplement: Supplementary file 1 [file DataSheet_1.zip › BLA/Figure S1.DOCX]
