# Supplementary material for: Floral Scent Composition and Fine-Scale Timing in Two Moth-Pollinated Hawaiian Schiedea (Caryophyllaceae)
Source: Front Plant Sci. 2020 Jul 21;11:1116. doi: 10.3389/fpls.2020.01116 (PMC7385411; doi:10.3389/fpls.2020.01116)

## Supplementary Figure S2

Grouping of volatile emissions patterns for *Schiedea kaalae* (5 plants, green, right) and *S. hookeri* (3 plants, purple, left) measured by PTR-MS. Light/dark cycles in the growth chamber over 2-4 d for each of the 8 plants are indicated by dark and light bands (top row). Relative emission rates per flower are represented by colors from black to yellow (low to high, scale on the right) and are scaled by the maximum emission rate per flower of each ion. This maximum is indicated by colors from black to white (low to high) on the sidebar (left). Tentative identifications are given on the right for each ion (Table 3). On the left, ions are clustered by their scaled time series using WPGMA hierarchical clustering of Pearson distances. The clustering is separated into 11 groups by cutting across the dendrogram at a uniform height. Compounds are often represented by multiple fragment ions, indicated with asterisks, and a particular mass can show contributions from more than one compound or fragment, indicated by slashes.


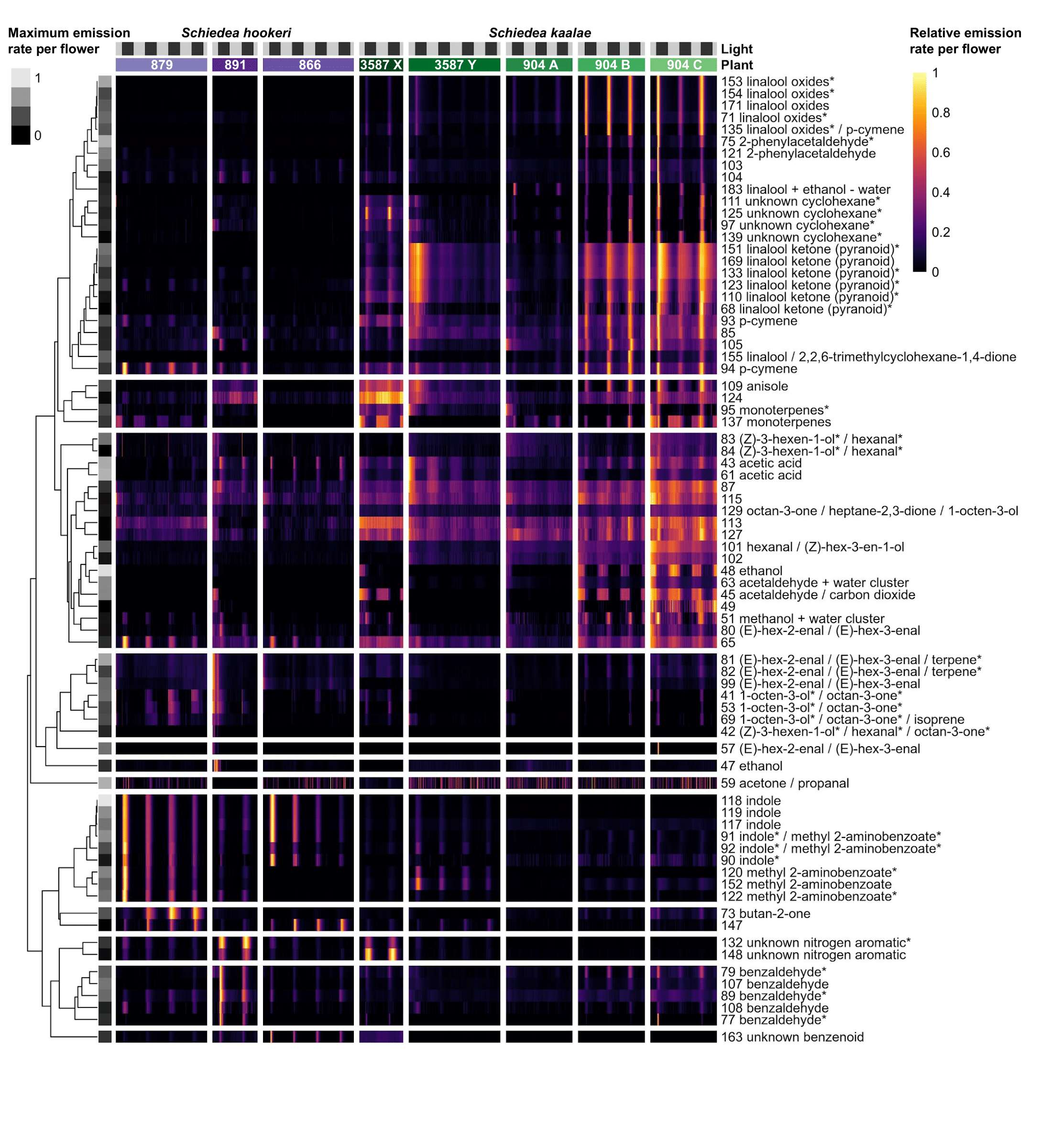

Supplement: Supplementary file 1 [file DataSheet_1.zip › BLA/Figure S2.DOCX]
