# Supplementary material for: Floral Scent Composition and Fine-Scale Timing in Two Moth-Pollinated Hawaiian Schiedea (Caryophyllaceae)
Source: Front Plant Sci. 2020 Jul 21;11:1116. doi: 10.3389/fpls.2020.01116 (PMC7385411; doi:10.3389/fpls.2020.01116)
Supplement: Supplementary file 1 [file DataSheet_1.zip › BLA/Methods S1.DOCX]

## Supplementary Methods S1

Each PTR-MS sampling session lasted two to four days and involved three plants. For each of the three sessions, three flowering plants were selected from the greenhouse, watered in the morning, and placed in a growth chamber (the UCI Fluxtron) at 23 °C and 60% relative humidity. This temperature is similar to the mean monthly temperature (mean ± SD from 1968 - 1976: 24 ± 2 °C) at a weather station 7 km away and 100 m lower than the ‘Ēkahanui Gulch site where pollinator observations were conducted (weather station Lualualei TWR NL 803.1, 21.4833° N, 158.1333° W, 458 m asl, data from the Global Historical Climatology Network, Menne et al., 2012). Plants received about 450 µmol·m^-2^·s^-1^ PAR, measured at the top of the inflorescence, from LED lights (LumiGrow Pro, LumiGrow, Emeryville, California, USA). This light level matches sunfleck conditions in Hawaiian mesic forest understory (Pearcy 1983). The photoperiod was 12 h light : 12 h dark, with immediate light transitions at 05:00 and 17:00 PST. For comparison, day length on Oʻahu ranges from 11 to 13 h throughout the year. Because sampling occurred at different times of year, these fixed times in the growth chamber were offset 0 - 1.75 h from the ambient photoperiod in the greenhouse. Several flowering inflorescences from each plant were placed in nylon-6 oven bag enclosures (40.6 cm x 44.4 cm). A fourth, empty enclosure was used as a blank sample. Each enclosure received dry zero air at the bottom of the enclosure from a zero-air generator (total of 12 L/min of air distributed among the 4 enclosures). Air was sampled from the top of the enclosure and 7 m long, 0.4 cm ID PFA sampling lines were heated at 50 °C. The enclosure was sealed with a tie around the peduncle, input and sampling lines, and thermocouple. Relays switched gas flow to the PTR-MS between the four enclosures, sampling the output flow from each plant or blank enclosure every 16 min in 4-min sampling blocks. This rotating sampling approach provides higher throughput compared to systems that sample one plant at a time. Input and output flows from each enclosure were monitored. An Ionicon PTR-Time-of-Flight-MS 1000 ultra (Ionicon Analytik, Innsbruck, Austria) was used to monitor masses from 33-205 *m/z* (mass/charge ratio) and the signal at each mass was averaged and recorded at 1 s intervals. The PTR-MS inlet flow was 140 standard cubic centimeters per minute, and the drift tube operated at 2.8 mbar, 60 °C, and 600 V.

We examined the fragmentation patterns of nine reference compounds to aid in identifying compounds in the PTR-MS spectra. Reference compounds were selected based on GC-MS results and commercial availability. We measured three terpenes (α-phellandrene, α-pinene, (−)-linalool), four aliphatic compounds (oct-1-en-3-ol, octan-3-one, hexanal, (Z)-hex-3-en-1-ol), and two benzenoids (benzaldehyde and indole). The fragmentation patterns for terpenes and aliphatics change with the energy level E/N (Maleknia *et al.,* 2007; Pang 2015; Kari *et al.*, 2018). For each compound, 100 μL liquid standard or c. 20 mg solid standard was added to a 2 mL GC vial, sealed, punctured with 1 cm of 1.5 mm ID teflon tubing, and placed in a 0.5 L glass jar. Zero air was pumped into the jar at 5 L/min and outflow split between an exhaust tube and the PTR-MS inlet. Spectra were averaged over 10 min after a 3 min equilibration period, and measurements alternated between standards and the empty jar washed in methanol to prevent carryover. If an ion’s signal rose twofold over the baseline, it was included in the spectrum for that compound. Under the PTR-MS conditions described, all reference compounds except indole underwent some level of fragmentation (Supplementary Figure S1). The terpenes α-phellandrene, α-pinene, and (−)-linalool showed typical fragmentation patterns (Tani *et al.,* 2003; Maleknia *et al.,* 2007; Misztal *et al.,* 2012; Tani 2013), each showing a major fragment ion at *m/z* 81. The aliphatics oct-1-en-3-ol, octan-3-one, hexanal, and (Z)-hex-3-en-1-ol and benzaldehyde also showed typical fragmentation (Buhr *et al.,* 2002; Maleknia *et al.,* 2007; Tasin *et al.,* 2012; Pang 2015).

Raw PTR-MS data and flow rates were processed using PTR-MS Viewer 3 (Ionicon Analytik, Innsbruck, Austria) and then in Igor Pro 7 (Wavemetrics, Inc., Lake Oswego, Oregon, USA) to calculate volatile fluxes from the difference between signals in the blank and plant enclosures. For analysis we rounded the *m/z* (mass-to-charge ratio) to integer values due to resolution limits of the instrument. Volatile fluxes were standardized to a per flower measure by dividing by the number of open flowers. We did not attempt quantitative calibration of volatile fluxes. Daily post-illumination bursts (on the order of minutes) of oct-1-en-3-ol and green leaf volatiles (C_6_ alcohols and alkenes) were observed in all *S. hookeri* inflorescences (unlike *S. kaalae*, *S. hookeri* inflorescences produce 1-12 mm long leaf-like bracts that could contribute to these emissions; Wagner et al.*,* 2005). Green leaf volatiles are known to be emitted transiently when photosynthesis is halted (Graus et al.*,* 2004), particularly during recovery from plant stress (Jud et al.*,* 2016), and so for analysis we excluded all data from the first 4-min sampling period following the light-to-dark transitions.

Tentative identities (molecular ions, fragment ions, or combinations of unresolved ions) of each *m/z* value were established by comparison to molecular weights of compounds detected by GC-MS in each species and time period and to published and experimental measurement of reference compounds (Supplementary Figure S1). Overlap of two compounds of a particular mass occurred for the six- and eight-carbon alcohols and aldehydes and for the terpenes, but many other compounds yielded ions with unique masses. Overlap of ions between different compounds occurred either due to isomeric product ions, or the inability of the PTR-MS instrument to distinguish similar masses. The overlap in molecular and fragment ions complicates PTR mass spectral analysis of mixtures (Pang 2015), and here we attempt only to distinguish compounds with identical or overlapping fragments when they produce a separate unique ion. In all other cases we indicate ambiguity in assignment with a slash between the potential contributors.

### References

Buhr, K., van Ruth, S., and Delahunty, C. (2002). Analysis of volatile flavour compounds by proton transfer reaction-mass spectrometry: fragmentation patterns and discrimination between isobaric and isomeric compounds. *International Journal of Mass Spectrometry* 221, 1–7. doi:[10.1016/S1387-3806(02)00896-5](https://doi.org/10.1016/S1387-3806(02)00896-5).

Graus, M., Schnitzler, J.-P., Hansel, A., Cojocariu, C., Rennenberg, H., Wisthaler, A., et al. (2004). Transient release of oxygenated volatile organic compounds during light-dark transitions in grey poplar leaves. *Plant Physiology* 135, 1967–1975. doi:[10.1104/pp.104.043240](https://doi.org/10.1104/pp.104.043240).

Jud, W., Vanzo, E., Li, Z., Ghirardo, A., Zimmer, I., Sharkey, T. D., et al. (2016). Effects of heat and drought stress on post-illumination bursts of volatile organic compounds in isoprene-emitting and non-emitting poplar. *Plant Cell Environ* 39, 1204–1215. doi:[10.1111/pce.12643](https://doi.org/10.1111/pce.12643).

Kari, E., Miettinen, P., Yli-Pirilä, P., Virtanen, A., and Faiola, C. L. (2018). PTR-ToF-MS product ion distributions and humidity-dependence of biogenic volatile organic compounds. *International Journal of Mass Spectrometry* 430, 87–97. doi:[10.1016/j.ijms.2018.05.003](https://doi.org/10.1016/j.ijms.2018.05.003).

Kim, S., Thiessen, P. A., Bolton, E. E., Chen, J., Fu, G., Gindulyte, A., et al. (2016). PubChem Substance and Compound databases. *Nucleic Acids Res* 44, D1202–D1213. doi:[10.1093/nar/gkv951](https://doi.org/10.1093/nar/gkv951).

Menne, M. J., I. Durre, R. S. Vose, B. E. Gleason, and T. G. Houston. (2012). An overview of the Global Historical Climatology Network-Daily Database. *J. Atmos. Oceanic Technol.* 29, 897–910. [doi:10.1175/JTECH-D-11-00103.1](https://doi.org/10.1175/JTECH-D-11-00103.1).

Maleknia, S. D., Bell, T. L., and Adams, M. A. (2007). PTR-MS analysis of reference and plant-emitted volatile organic compounds. *International Journal of Mass Spectrometry* 262, 203–210. doi:[10.1016/j.ijms.2006.11.010](https://doi.org/10.1016/j.ijms.2006.11.010).

Misztal, P. K., Heal, M. R., Nemitz, E., and Cape, J. N. (2012). Development of PTR-MS selectivity for structural isomers: monoterpenes as a case study. *International Journal of Mass Spectrometry* 310, 10–19. doi:[10.1016/j.ijms.2011.11.001](https://doi.org/10.1016/j.ijms.2011.11.001).

Pang, X. (2015). Biogenic volatile organic compound analyses by PTR-TOF-MS: calibration, humidity effect and reduced electric field dependency. *Journal of Environmental Sciences* 32, 196–206. doi:[10.1016/j.jes.2015.01.013](https://doi.org/10.1016/j.jes.2015.01.013).

Pearcy, R. W. (1983). The light environment and growth of C3 and C4 tree species in the understory of a Hawaiian forest. *Oecologia* 58, 19–25. doi:[10.1007/BF00384537](https://doi.org/10.1007/BF00384537).

Tani, A. (2013). Fragmentation and reaction rate constants of terpenoids determined by proton transfer reaction-mass spectrometry. *Environ. Control Biol.* 51, 23–29. doi:[10.2525/ecb.51.23](https://doi.org/10.2525/ecb.51.23).

Tani, A., Hayward, S., and Hewitt, C. N. (2003). Measurement of monoterpenes and related compounds by proton transfer reaction-mass spectrometry (PTR-MS). *International Journal of Mass Spectrometry* 223–224, 561–578. doi:[10.1016/S1387-3806(02)00880-1](https://doi.org/10.1016/S1387-3806(02)00880-1).

Tasin, M., Cappellin, L., and Biasioli, F. (2012). Fast direct injection mass-spectrometric characterization of stimuli for insect electrophysiology by proton transfer reaction-time of flight mass-spectrometry (PTR-ToF-MS). *Sensors (Basel)* 12, 4091–4104. doi:[10.3390/s120404091](https://doi.org/10.3390/s120404091).

Wagner, W. L., Weller, S. G., and Sakai, A. (2005). Monograph of *Schiedea* (Caryophyllaceae subfam. Alsinoideae). *Systematic Botany Monographs* 72, 1–169.
