# Supplementary material for: Floral Scent Composition and Fine-Scale Timing in Two Moth-Pollinated Hawaiian Schiedea (Caryophyllaceae)
Source: Front Plant Sci. 2020 Jul 21;11:1116. doi: 10.3389/fpls.2020.01116 (PMC7385411; doi:10.3389/fpls.2020.01116)
Supplement: Supplementary file 1 [file DataSheet_1.zip › BLA/Table S1.DOCX]

## Supplementary Table S1

Localities of *Schiedea* populations in this study (Wagner *et al.,* 2005). Collections of *S. hookeri* at Waiʻanae Kai were treated as a single population (WK) for this study. Figures are labeled with the population number only.

| **Species** | **Range** | **Location** | **Population collection number** |
| --- | --- | --- | --- |
| *S. hookeri* | Waiʻanae | Kaluaʻa Gulch, S of Puʻuhapapa | Weller and Sakai 879 (BISH, US) |
|  |  | Waiʻanae Kai, various locations. Ridge separating Waiʻanae Kai and Makaha Valley (Weller and Sakai 794, BISH), ridge separating Makaha and Waiʻanae Valleys (Weller and Sakai 866, US), below Kaʻala (Weller and Sakai 891, BISH, PTBG, US), gulch between the Makaha-Waiʻanae ridge and Puʻukalena (Weller and Sakai 899, US) | WK |
| *S. kaalae* | Waiʻanae | Kaluaʻa Gulch, S of Puʻuhapapa | Weller and Sakai 892 (US) |
|  |  | Pahole Gulch | Weller and Sakai 904 (BISH, PTBG, US) |
|  |  | E of Puʻukaua, near Puʻumaialau | Takeuchi 3587 (BISH) |
|  | Koʻolau Range | Makaua Valley (Hidden Valley) | Weller and Sakai 881 (BISH, PTBG, US) |

### *Reference*

Wagner, W. L., Weller, S. G., and Sakai, A. (2005). Monograph of *Schiedea* (Caryophyllaceae subfam. Alsinoideae). *Systematic Botany Monographs* 72, 1–169.
