# Supplementary material for: Floral Scent Composition and Fine-Scale Timing in Two Moth-Pollinated Hawaiian Schiedea (Caryophyllaceae)
Source: Front Plant Sci. 2020 Jul 21;11:1116. doi: 10.3389/fpls.2020.01116 (PMC7385411; doi:10.3389/fpls.2020.01116)
Supplement: Supplementary file 1 [file DataSheet_1.zip › BLA/Table S3.DOCX]

## ***Supplementary Table S3***

Diel (daily) changes in floral scent of *Schiedea kaalae* and *S. hookeri*. Protonated PTR-MS ions are listed with their mass-to-charge ratio (*m/z*), tentative identification(s) and fragment ions (*), average diel ratio (evening/day ratio) between 19:00 - 20:00 PST (2 - 3 h after dark) and 12:00 - 13:00 PST (5 - 4 h before dark), and grouping by start time (see Results): (M) morning, (A) afternoon, (D) dark, (+) a combination of two groups. Diel ratios > 2 are bolded to show which compounds had the strongest relative fluctuations. Slashes indicate contributions to the signal from more than one compound or fragment at that *m/z*. Compounds positively identified in the GC-MS data are given bold names; the others are tentatively identified using only PTR-MS. References: R = reference standard run on same instrument (see Supplementary Figure S1); S = ion present in both experimental and/or NIST 70 eV electron ionization spectra; G = no available reference but molecular ion of compound present in GC-MS data; 1 = Buhr et al. (2002); 2 = Gueneron et al. (2015).

| ***m/z*** | **Identification** | ***S. kaalae* diel ratio** | ***S. kaalae* group** | ***S. hookeri* diel ratio** | ***S. hookeri* group** | **Source** |
| --- | --- | --- | --- | --- | --- | --- |
|  | ***Aliphatics*** |  |  |  |  |  |
| 84 | **(Z)-hex-3-en-1-ol* / hexanal*** | 2.0 | - | **2.1** | - | R 1 / R 1 |
| 83 | **(Z)-hex-3-en-1-ol* / hexanal*** | 1.6 | - | **2.2** | - | R 1 / R 1 |
| 53 | **oct-1-en-3-ol* / octan-3-one*** | - | A | **61.3** | D | R / R |
| 41 | **oct-1-en-3-ol* / octan-3-one*** | **5.9** | A | **14.0** | D | R 1 / R |
| 69 | **oct-1-en-3-ol* / octan-3-one*** / isoprene | **10.2** | A | **4.8** | D | R 1 / R / 1 |
| 99 | **(E)-hex-2-enal / (E)-hex-3-enal** | **2.1** | - | 1.5 | - | G / G |
| 80 | **(E)-hex-2-enal / (E)-hex-3-enal** | 1.5 | - | **37.4** | D |  |
| 82 | **(E)-hex-2-enal / (E)-hex-3-enal** / terpene* | **2.1** | A | 1.6 | - |  |
| 129 | **octan-3-one / heptane-2,3-dione / oct-1-en-3-ol** | 1.0 | - | **2.3** | D | R 1 / / R 1 |
| 63 | acetaldehyde + water cluster | 0.6 | M | - | - |  |
| 61 | acetic acid | 0.5 | M | **7.8** | M+D |  |
| 43 | acetic acid | 1.3 | M | **11.7** | M+D | R / R |
| 59 | acetone / propanal | 1.3 | - | **9.9** | - |  |
| 73 | butan-2-one | 1.9 | A | **12.9** | A | 1 |
| 48 | ethanol | 0.2 | M | - | - |  |
| 101 | **hexanal / (Z)-hex-3-en-1-ol** | 0.8 | M | 1.0 | M | R 1 / R |
| 33 | methanol | 1.8 | - | 1.3 | - |  |
| 51 | methanol + water cluster | **6.8** | A | - | D |  |
| 144 | unknown cyclohexane | **6.6** | A | **4.1** | - |  |
| 139 | unknown cyclohexane* | **10.8** | A | 0.2 | - |  |
| 125 | unknown cyclohexane* | **9.4** | A | 0.2 | - | 2 |
| 111 | unknown cyclohexane* | **12.6** | A | **2.3** | - | 2 |
| 97 | unknown cyclohexane* | **9.1** | A | 0.4 | - | 2 |
|  | ***Benzenoids*** |  |  |  |  |  |
| 121 | **2-phenylacetaldehyde** | **9.8** | A | **7.8** | D | G |
| 75 | **2-phenylacetaldehyde*** | **6.1** | M+A | 0.6 | - |  |
| 109 | **anisole** | **3.2** | A | 0.6 | M |  |
| 108 | **benzaldehyde** | **10.8** | A | **91.0** | D | R |
| 107 | **benzaldehyde** | **3.0** | A | **70.4** | D | R |
| 89 | **benzaldehyde*** | 1.0 | - | **8.7** | D |  |
| 79 | **benzaldehyde*** | **3.9** | A | **29.2** | D | R |
| 77 | **benzaldehyde*** | - | D | - | D |  |
| 119 | **indole** | **12.5** | A | **201.1** | A | R |
| 118 | **indole** | **53.3** | A | **155.2** | A | R |
| 117 | **indole** | 1.6 | A | **36.2** | A | R |
| 90 | **indole*** | 1.9 | A | **241.6** | A |  |
| 92 | **indole* / methyl 2-aminobenzoate*** | **6.7** | A | **86.7** | A | S |
| 91 | **indole* / methyl 2-aminobenzoate*** | **9.9** | A | **131.2** | A | R |
| 152 | **methyl 2-aminobenzoate** | **6.8** | A | **245.1** | A | M |
| 122 | **methyl 2-aminobenzoate*** | **9.9** | A | **97.1** | A |  |
| 120 | **methyl 2-aminobenzoate*** | **31.4** | A | **84.4** | A | S |
| 163 | **unknown benzenoid** | 0.9 | M | **10.4** | D | G |
| 148 | unknown nitrogen aromatic ^1^ | **21.5** | A | **54.8** | A |  |
| 132 | unknown nitrogen aromatic* | **29.6** | A | **441.7** | A |  |
|  | ***Monoterpenes*** |  |  |  |  |  |
| 81 | **(E)-hex-2-enal / (E)-hex-3-enal** / terpene* | 1.5 | M+A | 1.5 | M+A | R / R / |
| 155 | **linalool / 2,2,6-trimethylcyclohexane-1,4-dione** | **4.3** | A | 0.0 | - | G |
| 183 | **linalool** + ethanol - water | **123.8** | A | 0.0 | - |  |
| 169 | **linalool ketone (pyranoid)** | **2.8** | A | 0.4 | - | G |
| 151 | **linalool ketone (pyranoid)*** | **3.0** | A | - | D | S |
| 133 | **linalool ketone (pyranoid)*** | **2.5** | A | **9.7** | D | S |
| 123 | **linalool ketone (pyranoid)*** | **3.8** | A | **8.9** | D |  |
| 110 | **linalool ketone (pyranoid)*** | **3.7** | A | 1.3 | - |  |
| 68 | **linalool ketone (pyranoid)*** | **10.2** | A | **24.8** | - |  |
| 171 | **linalool oxides** | **14.8** | A | 0.0 | - | G |
| 154 | **linalool oxides*** | **16.6** | A | 1.9 | - | S |
| 153 | **linalool oxides*** | **16.4** | A | 0.0 | - | S |
| 71 | **linalool oxides*** | **6.1** | A | 1.5 | - | S |
| 135 | **linalool oxides* / p-cymene** | **26.9** | A | **376.3** | D | S |
| 137 | monoterpenes | 1.1 | M+A | 0.3 | M | R |
| 95 | monoterpenes* | **2.1** | M | **2.7** | - |  |
| 94 | **p-cymene** | **2.6** | A | **5.0** | D |  |
| 93 | **p-cymene** | 1.9 | A | **3.4** | D | R |
|  | ***Unknowns*** |  |  |  |  |  |
| 104 | unknown 103 | **4.0** | D | **10.3** | D |  |
| 103 | unknown 103 | **2.5** | M+D | **4.0** | D |  |
| 105 | unknown 105 | **2.9** | A | **5.9** | D |  |
| 115 | unknown 115 | 0.9 | M | 0.9 | M |  |
| 87 | unknown 115* | 0.9 | M | 1.9 | - |  |
| 116 | unknown 116 | 1.4 | - | **8.9** | D |  |
| 65 | unknown 116* | 1.0 | M | **5.1** | D |  |
| 147 | unknown 147 | **6.0** | D | **40.5** | D |  |
| 199 | unknown 199 | **17.7** | D | 0.4 | - |  |

^1^ *m/z* 148 and 132 are highly correlated across plant time series. All of the NIST EI spectra with peaks at the corresponding deprotonated *m/z* [are nitrogen-containing aromatics.](https://doi.org/10.1111/jipb.12511)

### *References*

Buhr, K., van Ruth, S., and Delahunty, C. (2002). Analysis of volatile flavour compounds by proton transfer reaction-mass spectrometry: fragmentation patterns and discrimination between isobaric and isomeric compounds. *International Journal of Mass Spectrometry* 221, 1–7. doi:[10.1016/S1387-3806(02)00896-5](https://doi.org/10.1016/S1387-3806(02)00896-5).

Gueneron, M., Erickson, M. H., VanderSchelden, G. S., and Jobson, B. T. (2015). PTR-MS fragmentation patterns of gasoline hydrocarbons. *International Journal of Mass Spectrometry* 379, 97–109. doi:[10.1016/j.ijms.2015.01.001](https://doi.org/10.1016/j.ijms.2015.01.001).
